# Attractin Participates in Schizophrenia by Affecting Testosterone Levels

**DOI:** 10.3389/fcell.2021.755165

**Published:** 2021-11-11

**Authors:** Nan Li, Shuzhan Gao, Shuang Wang, Sijie He, Jiayin Wang, Luqingqing He, Dongya Jiang, Yun Stone Shi, Jianguo Zhang, Yuan Gu, Tian Chen, Mingjun Kong, Xijia Xu, Qingshun Zhao

**Affiliations:** ^1^Department of Psychiatry, Nanjing Brain Hospital, Medical School, Nanjing University, Nanjing, China; ^2^Model Animal Research Center, Medical School, Nanjing University, Nanjing, China; ^3^Department of Psychiatry, Affiliated Nanjing Brain Hospital, Nanjing Medical University, Nanjing, China; ^4^BGI-Shenzhen, Shenzhen, China

**Keywords:** ATRN, schizophrenia, zebrafish, behavior, testosterone

## Abstract

Attractin (ATRN) is a widely expressed glycoprotein that is involved in energy homeostasis, neurodevelopment, and immune response. It is encoded by a gene spanning 180 kb on chromosome 20p13, a region previously implicated in schizophrenia by linkage studies. To address a possible role of *ATRN* in disorders of the central nervous system, we created an *atrn* knockout zebrafish line and performed behavioral tests. Adult *atrn^–/–^* zebrafish exhibited more pronounced attack behavior relative to wild-type control zebrafish in a tracking analysis. Biochemical analysis revealed elevated testosterone levels in *atrn^–/–^* zebrafish. At the gene expression level, we noted an upregulation of *cyp51* and *hsd17b7*, key proteins in testosterone synthesis in the brains of both adult and larvae of *atrn^–/–^* zebrafish. In order to further elucidate the relationship between testosterone and behavioral syndromes, we then compared testosterone levels of 9,008 psychiatric patients and 247 healthy controls from the same catchment area. Of all subjects examined, male subjects with schizophrenia exhibited lower testosterone levels compared with controls. In contrast, female subjects with a diagnosis of schizophrenia or bipolar disorder featured higher testosterone levels than did same sex controls. Purposeful sampling of extreme groups showed reduced *ATRN* expression in a subset of these subjects. Finally, we identified 14 subjects with *ATRN* mutations. All of whom displayed abnormal testosterone levels. In summary, the interplay of *ATRN* and testosterone may help to explain sexual dimorphisms in selected behavioral phenotypes.

## Introduction

Attractin is a member of the cell adhesion and guidance family of proteins containing a CUB domain, and EGF domain, and kelch repeats (Wrenger et al., 2006). It is widely expressed in humans and occurs in two isoforms of 175 and 200 kDa, encoded by the *ATRN* gene on chromosome 20p13 (Tang et al., 2000). Attractin, the membrane-bound isoform, with sequence similarity to the mouse mahogany protein, forms a receptor controlling obesity (Kuwamura et al., 2002). The secretary isoform, a dipeptidyl peptidase IV/CD26-like enzyme, is expressed in human peripheral blood monocytes and has been shown to play important roles in cell-mediated immunity and neurodevelopment (Duke-Cohan et al., 1998; Paz et al., 2007).

Insight into the physiological function of Attractin has been obtained mostly from the study of rodent *Atrn* mutants. Rats with a null mutation in *Atrn* exhibit a tremor from around 3 weeks of age (Kuramoto et al., 2002; Shahrour et al., 2017) and have been named zitter rats. Mice deficient in *Atrn* feature changes in pigmentation due to disruption of the Mc1r–Agouti signaling pathway. Specifically, interference with Atrn suppresses the normal effects of Agouti on pigment production, leading to production of yellow pigment, the development of juvenile obesity, and hyperinsulinemia (Gunn et al., 2001). In addition, *Atrn* mutant mice show reduced body weight and adiposity (Kuramoto et al., 2002). ATRN is a co-receptor for Agouti regulation of pigmentation and obesity in MC1R and MC4R signaling pathways (Barsh et al., 2002). Mutant mice have also shown more nocturnal activity than wild-type mice in behavioral studies (Kuwamura et al., 2002). In 2012, Kohn studied the genetics of schizophrenia in a large inbred Arab–Israeli pedigree and found evidence for linkage on chromosome 20p13 (Teltsh et al., 2013). This locus harbors four strong candidate genes for schizophrenia: attractin (*ATRN*), pantothenate kinase 2 (*PANK2*), oxytocin (*OXT*), and arginine–vasopressin (*AVP*). Besides, He found that cDNA FLJ58441 is highly similar to attractin, and it has a high expression level in schizophrenia patients (Xu et al., 2018). Though multiple functions have been discovered, little is known about the role of attractin and the molecular mechanisms in mental disorders.

Mental disorders refer to a wide range of illnesses that affect perception, thinking, mood, and behavior due to poor mental health conditions, including schizophrenia, bipolar disorder, etc., In the psychiatric clinic, patients with mental disorders have their own behavior patterns, and often, these abnormal behaviors eventually lead to patients seeking medical treatment. Mental disorders have complex causes, among which genetic factors are triggering factors, and environmental factors are promoting factors (Piccin and Contarino, 2020). Motor behavior includes various movements from involuntary twitches to target actions, and involves every part of the body in an environment from individual actions to social interactions (Minassian et al., 2017), and motor behaviors can emerge from a mix of interacting factors (Wayner et al., 1970). Including genetic factor and hormone level. Some of the hormones are thought to directly correlate to mental disorder, but the inner mechanism is still unclear. For example, the results showed that schizophrenia patients had significantly increased testosterone levels when compared with healthy control subjects (Huang et al., 2021). Another research has found that mean total testosterone serum levels were significantly higher in bipolar disorder patients in comparison with major depressive disorder patients (Flores-Ramos et al., 2020). There is evidence that hypogonadism (lack of testosterone) is related to poor mental health outcomes and is a risk factor for depression and anxiety (Almeida et al., 2004; Shores et al., 2005). It is still unknown how neuroendocrine hormones and the phenotype of mental disorders are related, and the underlying mechanism is still unclear.

Among the multimodel animals, zebrafish has become one of the preferred model animals for studying human diseases due to its *in vitro* fertilization, early embryonic transparency, and easy genetic manipulation (Myers et al., 1986; Metcalfe and Westerfield, 1990; Sampath et al., 1998; Miklosi and Andrew, 2006; Morris, 2009; Shang et al., 2015). In addition, the genome of zebrafish is highly conserved with humans, and about 84% of human pathogenic genes have homologous genes on zebrafish (McCammon and Sive, 2015). Zebrafish exhibits complex and extensive behavioral patterns (Cachat et al., 2010), including social, anxiety, aggression, learning, and memory, cluster behavior, etc., which can be used in large-scale behavioral studies (Miklosi and Andrew, 2006; Jones and Norton, 2015; Rodrigues et al., 2016; Gupta et al., 2018; Caldwell et al., 2019).

In this study, we constructed an *atrn* mutant zebrafish line using the CRISPR/Cas9 technology (Komor et al., 2017; Liu et al., 2018). The tracking behavior analysis of adult and larvae of different genotypes with different genders was performed. The velocity of the *atrn^–/–^* adult male zebrafish and larvae is increased, the testosterone content is also increased, and the cholesterol synthesis pathway is activated. It is found that Atrn is involved in the cholesterol-related synthesis pathway, which affects the testosterone levels in zebrafish, changes the hormone levels, and causes changes in behavioral levels. In addition, there is a strong correlation between abnormal testosterone levels in schizophrenia patients with *ATRN* mutations.

## Materials and Methods

### Ethics Statement

The breeding and experimental protocols involved in using zebrafish were approved by the IACUC of the Model Animal Research Center, Nanjing University. All methods were performed in accordance with the relevant guidelines and regulations.

The studies involving human participants were reviewed and approved by the local ethics committee of the Affiliated Nanjing Brain Hospital, Nanjing Medical University (2017-KY017). Written informed consent to participate in this study was provided by the legal guardian/next of kin of the participants.

### Microinjection of Cas9 and sgRNAs Into Zebrafish Embryos

Two sgRNAs were designed according to the website.^1^ The sequences of sgRNAs are **GAUAAAAUCUACAUGUACGG**GUU UUAGAGCUAGAAAUAGCAAGUUAAAAUAAGGCUAGUCC GUUAUCAACUUGAAAAAGUGGCACCGAGUCGGUGCUU UUUUU (atrn-sgRNA1), **GGAGGGAAGAUUGACUCCAC**GU UUUAGAGCUAGAAAUAGCAAGUUAAAAUAAGGCUAGU CCGUUAUCAACUUGAAAAAGUGGCACCGAGUCGGUGC UUUUUUU (atrn-sgRNA2). Two sgRNAs were mixed with Cas9 mRNA and microinjected into the embryos at one-cell stages. The microinjection amount was 1 nl of solution containing 0.05 ng of two sgRNAs and 0.25 ng of Cas9 mRNA. When screening the mutation, the primers were designed to identify the genotypes. atrn −4 F: 5′-CAGGATAAAATCTACATGTA-3′; atrn −4 F(34): 5′-CAGGATAAAATCTACACGGA-3′; atrn −22 F: 5′-TAAAATCTACATGTACGGAG-3′; atrn −22 F(34): 5′-TAAAATCTACATGTAGGAAA-3′; atrn R: 5′-ATATTGTACTG CTGCACTTG-3′.

### Whole-Mount *in situ* Hybridization

Digoxigenin-UTP-labeled antisense RNA probes were synthesized *in vitro* using a linearized plasmid or PCR product as template. The template of atrn probe was amplified with the following primers: 5′-CCTCTCAAAGCTGGATGACATTA AAC-3′ (forward) and 5′-CACACTTCTCTCCATCTGCATAC-3′ (reverse). Whole mount *in situ* hybridization (WISH) on examining the expression patterns of atrn was performed as described previously (Yue et al., 2018). The embryos were raised to adults as founders. The F1 mutant zebrafish were screened using the method as described before (Kantari et al., 2007). Briefly, F1 generation zebrafish were genotyped by the PCR method using the genomic template prepared from the caudal fins clipped from zebrafish older than 6 weeks with a commercial kit (YSY, China). The primers used for amplifying the genomic sequences were 5′-TATGAGACTCGGTGCTGCAG-3′ and 5′-CCTAAAACATGTCCTGTACTGTATG-3′. The PCR conditions were 95°C for 2 min, 35 cycles (94°C for 30 s, 56°C for 30 s, and 72°C for 30 s), and a final extension of 10 min at 72°C. F1 mutant zebrafish carrying the atrn knockout allele were selected and inbred to get atrn null progeny (F2).

### Semiquantitative PCR

Real-time RT-PCR assay (qRT-PCR) was performed to examine the relative expression levels of the genes of interest. For the coding genes in this study, we used ABI Step One Plus (Applied Biosystems), as reported previously (Yue et al., 2018). Transcript levels of the examined coding genes were normalized to the gapdh mRNA level according to standard procedures. We extracted total RNA from at least 30 embryos, three brains or testis in each group using Direct-zol RNA Mini-Prep (Zymol Research). The venous samples of six healthy people and 20 patients suffering from mental disorder with a high testosterone level were extracted total RNA by using Direct-zol RNA Mini-Prep (Zymol Research). Zebrafish and human venous cDNA at indicated stages were prepared by HiScript II 1st Strand cDNA Synthesis (Vazyme). The PCR was performed as follows: 50°C for 2 min, 95°C for 10 s, 40 cycles (95°C for 5 s, 60°C for 20 s), 95°C for 15 s, 60°C for 30 s, 95°C for 15 s. The primers used are listed as below: cyp51-rt-F1: 5′-CACACGGAGAAACACACAACCAC-3′, cyp51-rt-R1: 5′-CT AACAATGTGCAACTGTAGTG-3′; hsd17b7-rt-F1: 5′-CCGA CCAAGCAAGATGGATCTTG-3′, hsd17b7-rt-R1: 5′-CTCCAT GAGCAGTTTATAAATGACC-3′, zgapdh-qF: 5′-CGCTGGCAT CTCCCTCAA-3′, zgapdh-qR: 5′-TCAGCAACACGATGGCTG TAG-3′. hATRN-RT-F1: 5′-TCCAGACGTAGAGAGCAACT TC-3′, hATRN-RT-R1: 5′-TTGTTGCCAAAACACGGCTC-3′, hGAPDH-Q-F1: 5′-CACTAGGCGCTCACTGTTCTC-3′, hGAPDH-Q-R1: 5′-CCAATACGACCAAATCCGTTGAC-3′.

### Live Imaging and Quantification

To observe the morphology, we treated embryos with 0.04 mg/ml MS-222 (Sigma) at room temperature for 5 min. Embryonic pictures were taken by an Olympus MVX10 microscope (Japan), and the photos were improved by Photoshop. The transgenic zebrafish lines Tg(*hb9*:eGFP), *atrn*^*nju*70^ and Tg(*olig2*:dsRed), and *atrn*^*nju*70^ were to confirm the development of motor neurons and oligodendrocytes. All confocal images were acquired using a Zeiss LSM880 confocal microscope. Then when measuring the weight and length of zebrafish, we treated them with 0.04 mg/ml MS-222 (Sigma), then blot the water, placed it in a Petri dish, measured it with an analytical balance, and recorded the data.

### Tracking Behavioral Analysis

Live video tracking of zebrafish larvae was performed by Zebralab Video-Track system (Viewpoint, France). The mature zebrafish (4 months postfertilization) were transferred to a box (well size: 11-cm length × 7-cm width × 7-cm height) with 400 ml of E3 medium, and larvae (aged 5 dpf, 6 dpf) were transferred to a 24-well culture plate with 1 ml of E3 medium. In larvae, the detection threshold value for movement was set at 2–20 mm/s. Locomotor activity was measured per 1 min, and videos were recorded for 5 or 10 min. In adult zebrafish, the detection threshold value for movement was set at 20–75 mm/s. Locomotor activity was measured per 1 min, and videos were recorded for 1 h.

### Mirror Attack Behavior

At 4 mpf, a mirror attack test was carried out using a box (well size: 11-cm length × 7-cm width × 7-cm height) with a one-sided mirror. Individual zebrafish was transferred to the apparatus with 400 ml of E3 medium. The movement was recorded for 10 min with data collection every 30 s. The detection parameter for movement was set at 20–75 mm/s, and the movement in the entire space and the movement in the mirror space were recorded.

### Whole-Transcriptome Deep Sequencing

Total RNA was isolated from the single brain of a 4-months postfertilization (mpf) wild-type and atrnnju70 zebrafish using TRIzol reagent (Invitrogen). The transcriptome sequencing was performed by Novel-Bio Bio-Pharm Technology Co. (Shanghai, China). Gene expression levels were quantified by RPKM (reads per kilobase of transcript per million mapped reads) arithmetic. The MapSplice software was used for data alignment, and EB-Seq arithmetic was used for the screening of differential expression genes.

### Testosterone ELISA Assay (Parameter, United States and Canada)

After the samples were separated, they were quick frozen with liquid nitrogen, 250 μl of 1 × PBS was added in the ice, the tissue was sufficiently broken with a grinder, and it was centrifuged at 12,000 × *g* at 4°C for 1 min. The next specific steps follow the protocol. A standard curve was created using a computer software, mELISA, which is capable of generating a four-parameter logistic (4-PL) curve-fit.

### Measure Concentrations of Serum Testosterone

This study was approved by the Ethics Committee of the Department of Psychiatry, Affiliated Brain Hospital of Nanjing Medical University, Jiangsu, China. From 2018 to 2020, all men and women aged 20 and above, their pathological information, including testosterone content, clinical diagnosis, etc., were all used for analysis. All individuals (including 3,588 males and 5,667 females) were required to have an empty stomach at least 8 h before venous sampling. The method of Elecsys Testosterone II, based on the principles of competition, was applied to measure concentrations of serum testosterone. Serum testosterone samples were analyzed by electrochemiluminescence using a Cobas e601 analyzer in the clinical laboratory of the Affiliated Nanjing Brain Hospital, Nanjing Medical University, and the kit was provided by Roche Diagnostics GmbH.

### Statistical Analyses

Experiments were performed two or three times independently. Data are shown as mean ± SD. Statistical analysis was carried out with GraphPad Prism 7 (GraphPad Software, La Jolla, CA, United States). Data were first tested for normality using the Kolmogorov–Smirnov’s test. If data sets exhibit normal distribution, we employed Student’s *t*-test for equal variances or Welch’s *t*-test for unequal variances. If data sets are found not to exhibit normal distribution, Mann–Whitney test was applied. Testosterone level differences between the patients with different mental disorders, and healthy controls were tested using Chi square. A value of *p < 0.05* (^∗^) was considered statistically significant, and *p* < 0.01 (^∗∗^) and *p* < 0.001 (^∗∗∗^) were considered very statistically significant.

## Results

### Zebrafish *Atrn* Is Mainly Expressed in the Central Nervous System During Early Embryonic Development and Ubiquitously Most Tissues in Adulthood

Previous studies have shown that *Atrn* mutant affects motor behavior in mice and rats (Khwaja et al., 2006; Sakakibara et al., 2008; Izawa et al., 2010; Cheng et al., 2014). To better understand the function of *Atrn* in neural behavior, we use zebrafish as a model animal. Colinear analysis showed that human *ATRN*, and mouse and zebrafish *atrn* were all colinear with *GFRA4* and *SLAC4A11*, indicating that zebrafish *atrn* and mamalian *atrn* are orthologous genes (Supplementary Figures 1A,B). Unlike human and rat *ATRN*, the zebrafish *atrn* gene only encodes a transmembrane form of protein, but it has two transcripts, one is 1,345 amino acids (aa) and the other is 1,383 aa (Sun et al., 2017), both of them containing crucial domains.

The developmental expression of *atrn* mRNA was examined by semiquantitative PCR relative to *actb2* as an endogenous control (Supplementary Figure 1C). From zygote to dome stage, *atrn* mRNA was widely expressed in animal poles (Sun et al., 2017), and maternal *atrn* mRNA was decreasing at this stage. By 24 h postfertilization (hpf), the zygote *atrn* mRNA was detected extensively in the head. This result suggests that Atrn may be involved in the development of the nervous system. In adult zebrafish, a high expression of *atrn* was mainly found in the brain, eyes, testes, and ovaries (Supplementary Figure 1D), in line with the widespread expression pattern of *ATRN* in human tissues (Tang et al., 2000).

### Knockout of *atrn* Does Not Affect Embryo Development, Maturation, and Fertility

**FIGURE 1:**
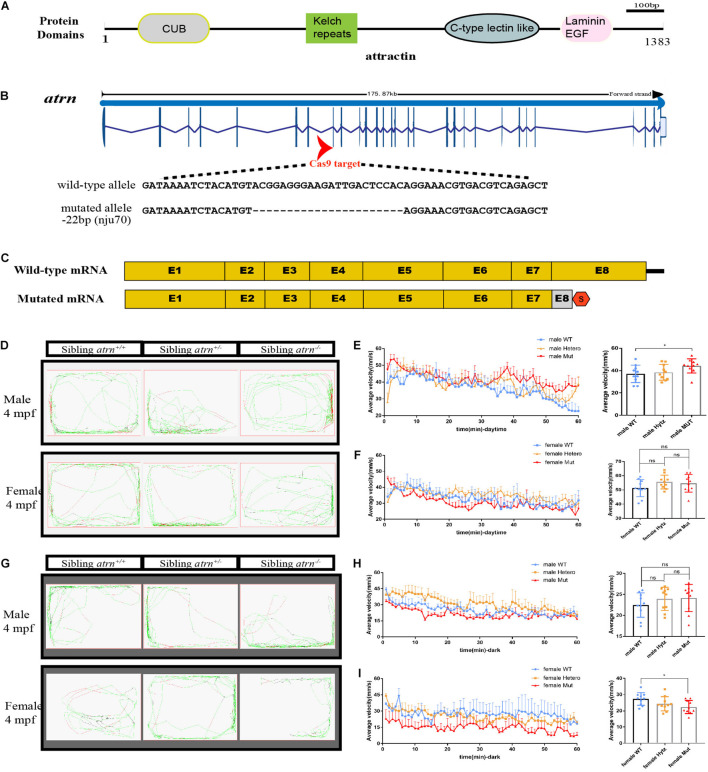
Generation of *atrn* mutation in zebrafish by CRISPR-Cas9 strategy, and the average velocity of the male mutant is faster than the wild type during the day, but in the dark, the female mutant likes to stay quiet. **(A)** Zebrafish attractin has the CUB domain, kelch-type beta propeller, C-type lectin-like domain, and laminin EGF domain. **(B)** Exon 8 is the target for CRISPR/Cas9 gene editing in zebrafish *atrn*. The CRISPR/Cas9-induced mutation (22-basepair deletion) in *atrn* is shown in annotated *atrn* mutant sequences. **(C)** The mutated *atrn* mRNA with PTC was predicted to encode truncated protein. **(D)** Locus diagram of male and female in sibling *atrn*^+^, sibling *atrn^+/–^*, and sibling *atrn^–/–^* groups at 4 mpf during daytime. Average velocity in 1 h was recorded in three genotype groups at 4 mpf. At 10:00–14:00, we analyzed the tracking data of male **(E)** and female **(F)** three-genotype groups; the average velocity of the male mutant is faster than the male wild type, but there is no significance among the three female groups. **(G)** Locus diagram of male and female in sibling *atrn*^+^, sibling *atrn^+/–^*, and sibling *atrn^–/–^* groups at 4 mpf at night. At 00:00–4:00, we analyzed the tracking data of male and female three genotype groups. The average velocity of the female mutant is slower than the male wild type **(H)**, but there is no significance among the three male groups in the night **(I)**. *N* = 10. Data are shown as mean ± SD; *p < 0.05; **p < 0.01; ***p < 0.001.

*Atrn* spans a total of 29 exons and encodes a protein with a conserved CUB domain, kelch repeats, C-type lectin-like domain, and laminin EGF domain (Figure 1A). To further analyze the function of Atrn in zebrafish, we designed two sgRNAs to target the eighth exon (Figure 1B). After screening, we obtained two *atrn* mutant lines, *nju70* and *nju71*, with deletion of 22 bases and 4 bases in the genomic sequence, respectively. The protein product prediction indicates that both encode nonfunctional truncated proteins (Figure 1C). The whole-mount *in situ* hybridization results showed that the expression of *atrn* in the maternal zygote (MZ) *atrn^–/–^* is reduced (Supplementary Figure 1B). The mutants were raised to adulthood and found to grow normally for breeding. Since the results produced by the two mutant lines were consistent, the *atrn*^*nju*70^ mutant line was chosen for the following study.

Morphology analysis of embryos produced by heterozygotes (*atrn*^*nju*70/+^) in-crosses showed no developmental defects in homozygous mutant embryos. When the embryos develop to adults, the MZ mutant is not different from the wild type (Supplementary Figure 1A). By crossing to the transgenic Tg (*hb9*: eGFP) and Tg (*olig2*: dsRed), we found that *atrn^–/–^* did not affect the development of motor neurons and oligodendrocytes (Supplementary Figure 1C).

### A Sexual Dimorphism Emerges in Locomotion, but *atrn^–/–^* Adult Zebrafish Exhibits Stronger Attack Behavior

Compared with wild-type (WT) littermates, *atrn^+/–^* and *atrn^–/–^* zebrafish showed reduced body weight in both genders at 4 months postfertilization (4 mpf) (Supplementary Figures 1D,E). This result is in line with studies in mice (Kuwamura et al., 2002). Body lengths of these mutant zebrafish were the same as WT littermates (Supplementary Figures 1F,G). Therefore, they appear slimmer (Supplementary Figure 1A).

We then analyzed their motion using ZebraLab (Viewpoint, French) during day (10:00–14:00) and night (0:00–4:00). A single adult zebrafish was placed in a box containing 400 ml of egg water and placed in Zebralab. The test time was 1 h, and the data were recorded every 1 min. The speed to stay inactive (black)/moderate motion (green) for adult fish was set to 25 mm/s, and the speed for moderate motion/large motion (red) is 70 mm/s. Ten males and females of different genotypes were tested each, and the analysis found that during the day, the trajectory distribution map of mutant males showed more large movements (Figure 1D). By counting the average velocity of each fish in each minute, and drawing the speed-time diagrams (Figures 1E,F), the average speed of the mutant males was significantly higher than that of the sibling wild type, and there were no differences among the three genotypes of females during the day. At night (Figure 1G), the average velocity of mutant females is significantly lower than that of the wild type, and they preferred to be quiet, while there is no significant difference between the three genotypes of males (Figures 1H,I). During the day, the behavior of mutant male zebrafish is consistent with the behavior of *Atrn* mutant mice.

**FIGURE 2:**
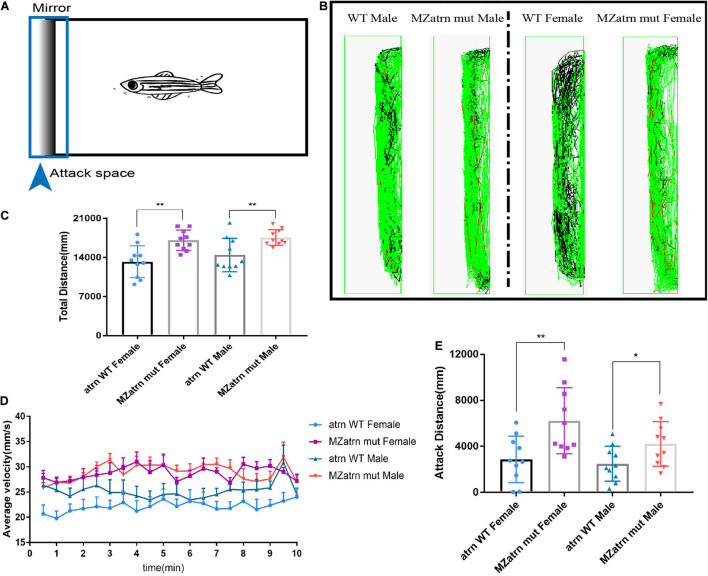
Mirror attack analysis indicated that the aggressive behavior of the *atrn^–/–^* adult zebrafish was significantly higher than that of the wild type. **(A)** Schematic diagram of the experimental design of the mirroring attack. **(B)** The mirror attack tracking pattern of the wild type (WT) and MZatrn mut with different gender in 10 min. Red: the swimming speed is quicker than 70 mm/s. Green: the swimming speed is between 25 and 70 mm/s. Black: the swimming speed is slower than 25 mm/s. **(C)** The total tracking distance in whole box space of MZatrn mut is higher than that of the wild type. **(D)** The velocity–time panel of four groups of zebrafish. **(E)** The tracking distance in mirror attack space of MZatrn mut is higher than that of the wild type. *N* = 10. Data are shown as mean ± SD; *p < 0.05; **p < 0.01; ***p < 0.001; ****p < 0.0001; ns, no significance (*p* > 0.05).

In order to confirm the effect of *atrn* knockout on zebrafish’s aggressive behavior, we designed a mirror attack experiment to test. A mirror was placed on the left side of the box, and ZebraLab was used to record the trajectory of the zebrafish within 10 min (Figure 2A). During the test, the movement distance of the female and male *atrn^–/–^* in the entire box was significantly higher than that of the wild type (Figure 2C), and the velocity–time distribution graph is also shown in Figure 2D. From the trajectory diagram of the zebrafish near the mirror (the attack space; the observer can determine whether it is a female or a male fish), *atrn^–/–^* zebrafish have more middle (velocity between 20–75 mm/s) and quicker (velocity higher than 75 mm/s) trajectories, while the wild type have more quiet trajectories (velocity lower than 20 mm/s) (Figure 2B). This confirms that the motility of *atrn^–/–^* is significantly more than that of the wild type. Finally, by counting the movement distance in the attack space, the attack movement distance of the *atrn^–/–^* is significantly higher than that of the wild type of the same sex (Figure 2E).

### Upregulation of Steroid Synthesis Pathway-Related Genes, Leading to Increased Levels of Testosterone in the Brain and Testis of the Mutant Males

**FIGURE 3:**
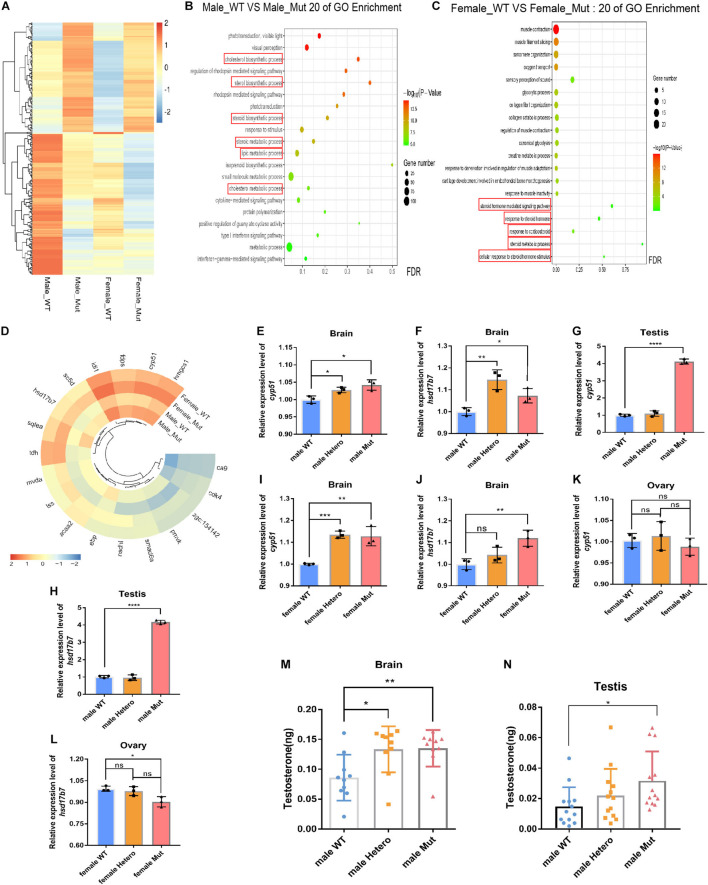
Transcriptome sequencing of the brain in the male and female *atrn* mutants, and steroid synthesis-related genes are upregulated in the mutant. **(A)** Heat map shows genes that are differentially expressed in the brain of wild type and mutants of different sexes. *N* = 3. **(B)** GO enrichment dot bubble of male wild type and male mutants indicate that GO terms related to steroid synthesis are affected. **(C)** GO enrichment dot bubble of female WT and female Mut indicate that GO terms related to steroid synthesis are affected. **(D)** Circular heatmap shows upregulation of genes related to steroid synthesis. qRT-PCR results for *cyp51* and *hsd17b7* gene expression in the brain **(E–J)** and testis of WT, heterozygotic (Hetero) and Mut zebrafish at 4 mpf, but there is no expression difference between the brain and ovary of the female adult zebrafish **(K,L)**. *N* = 9. Testosterone ELISA results show that the content of testosterone in the brain of the male mutant **(M)** and testis is significantly increased **(N)**. *N* = 10. Data are shown as mean ± SD; *p < 0.05; **p < 0.01; ***p < 0.001; ****p < 0.0001; ns, no significance (*p* > 0.05). Heatmap was plotted by http://www.bioinformatics.com.cn, an online platform for data analysis and visualization.

In order to understand the underlying mechanism of the effect of Atrn on behavior, brains were isolated from 4 mpf wild-type and mutant male and female adult zebrafish. Three brains were taken from each group and sent for transcriptome sequencing analysis (Figure 3A). The steroid synthesis pathway was enriched in results of the male and female mutant brains (Figures 3B,C; Kuwamura et al., 2002). The expression levels of related genes in the steroid synthesis pathway were upregulated, including *cyp51*, encoding the key enzyme for cholesterol synthesis, and *hsd17b7*, encoding the key enzyme for testosterone synthesis (Figure 3D).

Testosterone is a steroid hormone synthesized from cholesterol (Hong et al., 2004). About 90% of testosterone comes from Leydig cells, and the rest is produced in the adrenal cortex and other tissues (Geniole et al., 2017; Guan et al., 2019). In the Leydig cell cytoplasm, lanosterol acts as a precursor to cholesterol synthesis, catalyzed by 14α-demethylase, which is encoded by *cyp51* (Geniole et al., 2017). Cholesterol enters the mitochondria with the help of StAR protein, produces pregnenolone under the catalysis of P450scc, and then synthesizes testosterone under the catalysis of 17β-hydroxylase, which is encoded by *hsb17b7* (Forgacs et al., 2005; Oyola and Handa, 2017).

qRT-PCR results confirmed that the expression levels of *cyp51* and *hsd17b7* were significantly increased in the brain and testis of the male mutants (Figures 3E–H). In the brain of female mutants, the expression levels of *cyp51* and *hsd17b7* were also increased (Figures 3I,J), while in the ovaries, *cyp51* was unaltered (Figure 3K), and *hsd17b7* was decreased (Figure 3L). Testosterone ELISA Assay (Parameter, United States and Canada) was then used to test the testosterone content in brain tissues and gonads of male and female zebrafish of different genotypes, 10 samples per group. The levels of testosterone in the brain tissue of heterozygous and mutant males are significantly higher than those of the wild type (Figure 3M). Similarly, the testosterone content of mutant testis is higher than that of the sibling wild type (Figure 3N).

### An Increase in Average Velocity of *atrn^–/–^* Larvae on 6 dpf Matches an Increase in Overall Testosterone Content

**FIGURE 4:**
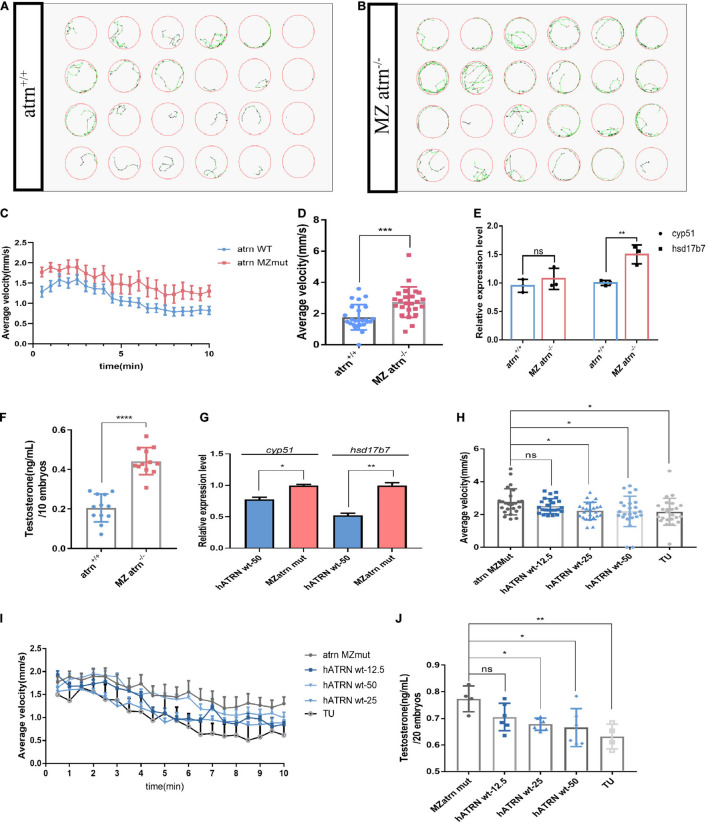
The movements of *atrn^–/–^* larvae are faster than the wild type due to higher testosterone concentration at 6 dpf, and human *ATRN* can rescue the phenotype of zebrafish *atrn^–/–^*. **(A)** The tracking pattern of the wild-type larvae. **(B)** The tracking pattern of the MZ *atrn^–/^*^–^ larvae. **(C)** Average swimming speed–time graph of *atrn* WT and MZmut larvae of 144 hpf in 10 min. **(D)** The average velocity of 24 WT and MZ *atrn^–/^*^–^ larvae during 10 min. Error bars, SEM. **(E)** qRT-PCR results indicate that the expression level of testosterone synthesis key gene *hsd17b7* in MZ *atrn^–/^****^–^*** larvae is increased. **(F)** Testosterone ELISA assay results show that the content of testosterone in the MZ *atrn^–/^****^–^*** is also increased. *N = 10*. **(G)** qRT-PCR results indicate that the expression level of *cyp51* and *hsd17b7* in treated MZmut larvae was rescued. **(H)** Average swimming speed–time graph of 6 dpf larvae injected with hATRN mRNA in 10 min. Error bars, SEM. **(I)** The average velocity of the control and treated larvae during 10 min. **(J)** Testosterone ELISA assay results show that the content of testosterone in larvae injected with hATRN mRNA was rescued. *N* = 20. Data are shown as mean ± SD; *p < 0.05; **p < 0.01; ***p < 0.001; ****p < 0.0001; ns, no significance (*p* > 0.05).

The adult male *atrn* MZmut exhibits more pronounced active behavior when compared with the wild type and feature higher testosterone levels (Figures 3E–H). The increase in testosterone levels was detected in both the brain and the testis. We sought to determine which source drives the change in locomotion. To answer these questions, we collected embryos produced by the wild type and the homozygous mutant in-crossing, and raised them to 28.5°C to 6 dpf. Twenty-four larvae were randomly selected and placed in a 24-well cell culture plate. One larva was placed in each well, and 1 ml of egg water was added. A 24-well cell culture plate was placed in Zebralab, the test time was 10 min, and data were recorded every 1 min. The speed of the inactive (black)/moderate motion (green) was set to 2 mm/s, and the speed of moderate motion/large motion (red) was 10 mm/s. The results show that the mutants are significantly more active than the wild type (Figures 4A,B). The speed–time diagram shows that during the entire test, the *atrn* MZmut had a significantly higher rate of movement than the wild type (Figure 4D). The average speed of each larva within 10 min was calculated, respectively (Figure 4C). At a statistical level, the average velocity of the mutants was significantly higher than that of the wild type (Figure 4D).

Similarly, wild-type and mutant embryos were collected at the same time period, and three samples in parallel were collected from each group, with 30 larvae per sample. The qRT-PCR results showed that the expression level of *cyp51* in the mutant was not different from that of the wild type, while the expression level of *hsd17b7* was significantly higher than that of the wild type (Figure 4E). At the same time, 12 genotypes of larvae were collected, each of which contained 10 larvae. After removing the egg water, 250 μl of 1 × PBS was added and milled. Testosterone ELISA Assay was used to test the overall testosterone content of one larva. The testosterone content in the mutant larvae was significantly higher than that of the wild type and heterozygotes (Figure 4F).

### Exogenous Testosterone Enhanced Larvae Motility

Does elevated testosterone have a direct effect on zebrafish locomotion? We exposed wild-type embryos to different concentrations of testosterone for treatment from the development of 24 hpf. The egg water was replaced every 24 h. The same concentration of testosterone was added and processed to 5 dpf. At this time, the zebrafish bladder was fully developed, and zebrafish were able to normally swim normally. The testosterone treatment group was divided into 1, 10, and 100 ng/ml. We then randomly selected 24 larvae from each group, placed them into a 24-well cell culture plate, and proceeded with the tracking analysis (Zebralab). The trajectory distribution chart clearly shows that the zebrafish juvenile group featured a significantly higher locus of movement than did the untreated group (Supplementary Figures 3A–D). Analysis of exercise rates after treatment with exogenous testosterone reveals an increase in average velocity of zebrafish juveniles. With the increase in testosterone concentration, the average velocity of larvae shows a downward trend in a dose-dependent manner (Supplementary Figures 3E,F). The above results indicate that the treatment with exogenous testosterone can mimic the phenotype observed in *atrn^–/–^*, but with the increase in testosterone concentration, due to its drug toxicity, it inhibits the behavior of zebrafish larvae.

### Human *ATRN* Rescues the Motor Phenotype in *atrn* Knockout Zebrafish

In the *atrn* MZmutant embryos, human wild-type *ATRN* (hATRN wt) was overexpressed, and we found that 25 and 50 pg of hATRN wt mRNA can efficiently reduce the swimming speed (Figures 4H,I). Similarly, hATRN wt mRNA also can rescue the higher testosterone level (Figure 4J) and the expression level of *cyp51*, *hsd17b7* in *atrn* MZmut zebrafish embryos (Figure 4G). The results indicated that human wild-type *ATRN* can significantly rescue the phenotype in *atrn* MZmut. These data support the interplay of testosterone and *ATRN* in shaping behaviors in the animal model.

### Clinical Impact of Circulating Testosterone Levels in Patients With Mental Disorders

Schizophrenia is characterized by disturbances in perception and thinking, in behavior, emotional, and social symptoms (including lack of motivation, lack of social interaction and apathy), and cognitive impairment (Brennan, 2011; Seibt et al., 2011). The above results have shown that *ATRN* has influence on the testosterone level and resulted to abnormal locomotion, which is similar to the positive symptoms (abnormally active), but the correlation between behavior and testosterone is still unknown. Some researchers have illustrated that more hormones can affect the development of mental disorder, for example, steroid hormones, especially testosterone, are related to emotional processing (Derntl et al., 2009; van Wingen et al., 2009).

The information of patients suffering from mental disorder in the hospital was collected to analyze the relationship between the expression level of *ATRN* and testosterone level. The clinical standard for testosterone content in the human body was determined in adults aged 20–49. The normal level for men is 8.64–29.00 nmol/L, and the normal level for women is 0.29–1.67 nmol/L. The normal testosterone level in adults aged over 50 is 6.68–25.70 nmol/L (men) and 0.10–1.42 nmol/L (women).

In light of the known testosterone effects on emotional processing, we postulated that levels of circulating hormone may serve as a surrogate marker. To put this information into perspective, both the expression level of ATRN and the testosterone level were both examined in subjects diagnosed with schizophrenia. Manufacturer data obtained from healthy adults served as a reference.

**FIGURE 5:**
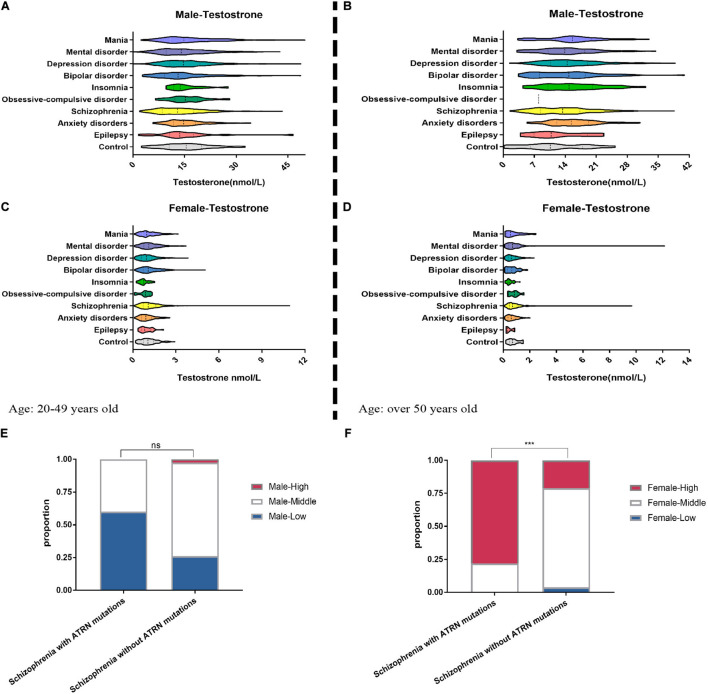
Clinical data show the abnormal serum testosterone level of mental disorder patients. We collected the medical records of patients with mental disorder in the hospital. Among 2,579 male patients **(A)** and 3,926 female patients **(C)** aged 20–49 years, the testosterone level in their serum was tested. Among the patients over the age of 50, the proportion of abnormal testosterone levels among 1,009 men **(B)** suffering from anxiety, insomnia, and depression was significantly increased **(E–G)**, but among 1,741 women **(D)** suffering from anxiety, insomnia, and depression, the proportion of abnormal testosterone levels is significantly reduced. **(E)** For five male patients with schizophrenia who are known to have *ATRN* mutations in the clinic, their lower serum testosterone levels are more than that of schizophrenia patients without *ATRN* mutations. **(F)** For nine female schizophrenia patients with *ATRN* mutations, serum testosterone level was higher than that of the schizophrenia patients without ATRN mutations. *Chi*-test (Fisher’s exact test). *p < 0.05; **p < 0.01; ***p < 0.001; ns, no significance *(p* > 0.05).

When stratified by age and gender, we counted 2,579 men aged 20–49 years, 3,926 women aged 20–49 years, 1,009 men aged over 50 years, and 1,741 women aged over 50 years. In the age group 20–49 years, men presenting with a diagnosis of schizophrenia were overrepresented relative to controls and featured lower testosterone levels (Figure 5A and Supplementary Table 1).

In contrast, women with schizophrenia were more likely to have higher testosterone levels. Likewise, women with a diagnosis of bipolar disorder also featured elevated testosterone levels (Figure 5C and Supplementary Table 2). In addition, among the test population over the age of 50, men with anxiety, insomnia, and depression tend to cluster in the category “high testosterone levels” (Figure 5B and Supplementary Table 3). Only a small group of women suffering from anxiety and insomnia exhibited higher testosterone levels (Figure 5D and Supplementary Table 4). Thus, a dimorphic pattern was observed primarily among patients with schizophrenia. Depending on gender, testosterone levels were either lower or higher than expected.

To establish the contribution made by *ATRN* expression level to testosterone levels and dimorphic effects, we then purposefully selected 14 patients with *ATRN* mutations who had been diagnosed with schizophrenia, including five men and nine women (Supplementary Table 5). At different points in time, testosterone levels were determined. We noted that in male patients, testosterone levels were still within the normal range or lower. In contrast, the testosterone levels of female patients were above the normal range (Supplementary Table 5). Moreover, male subjects with ATRN mutations were more likely to be classified as “low testosterone level.” Woman with ATRN mutations were more likely to be classified as “high testosterone level” (Figures 5E,F).

## Discussion

Hormones promote the pathophysiology of schizophrenia by increasing local dopamine synthesis and metabolism (Salilew-Wondim et al., 2015; Chen et al., 2019). Besides, many hormones affect the movement behavior of organisms, such as epilepsy, depression, etc. (Bodnar et al., 2018). Some mental disorders, such as schizophrenia, usually have abnormal behaviors. Previous researches have been reported that chromosome position where *ATRN* is located is highly related to schizophrenia (Teltsh et al., 2008). In this research, we used CRISPR/Cas9 technology to construct the *atrn^–/–^* zebrafish lines (Figure 1B). The tracking analysis suggests that different genders have different behavior and more aggressive attack behavior, indicating that it may affect the hormone levels in *atrn^–/–^* zebrafish. Testosterone ELISA Assay was used to detect whether the content in brain of mutant males was significantly higher than that of WT (Figures 3M,N). At the same time, it was also found that the testosterone content of the mutant was higher than that of the wild type in the testis of male zebrafish, but the testosterone content in the testis was not as high as that in the brain tissue. It was found that the testosterone content of *atrn* MZmut larvae was significantly higher than that of the wild type (Figure 4F). This result suggests that testosterone produced in brain tissue plays a more important role.

Sex steroids have long been implicated in the etiology of neurodevelopment disorders and may contribute to our understanding of sexual dimorphisms. In the present study, *atrn^–/–^* zebrafish lines were created and served to investigate behavioral phenotypes, and address hormone states and gene/protein expression (Zhao et al., 2021). With regard to brains of zebrafish, testosterone immunoassays indicated an excess of testosterone in mutant males relative to the wild type (Figures 3M,N). A similar pattern was observed in the testis and in larvae (Figure 4F). This result confirms that testosterone synthesis is affected in multiple tissues. In the early stages of development, starting at 4 hpf, primordial germ cells (PGC) undergo specialization, migration to the genital ridge, and proliferation (Kossack and Draper, 2019). The apoptosis-dependent transition from ovary to testis starts at 21–25 dpf and may last for several weeks. Only by 35 dpf has the sex of the gonads been determined, and sex-specific gametes are being produced in both the ovaries and testes (Wang et al., 2017; Luzio et al., 2021). As there is no mature testis in larvae, effects on locomotion of *atrn* MZmut are likely mediated by steroids originating from the brain. This observation further underscores the impact of downstream effects exerted by *atrn* on the nervous system.

There is a consensus that sex hormones play roles in anxiety-, trauma-, and stress-related disorders (Cover et al., 2014; Li and Graham, 2017). The potential for neuroactive steroids to participate in mood and cognitive regulation has fueled expectations of new treatment strategies (Sassarini, 2016). As for circulating testosterone levels, conflicting views exist on the existence of disease-specific changes in sex steroids (Belgorosky et al., 1989). Even so, most changes reported would appear to be minor (Lichtenstein et al., 2009). It should be emphasized that in the above studies, most of the changes are still within the normal range (Hong et al., 2004).

While the magnitude of clinical effects, thus, remains to be determined, and additional regulators of testosterone levels need to be taken into account, the behavior impact of mutated *atrn* warrants further study. Large-scale investigation may help identify common functional variants in the *ATRN* gene, predicting susceptibility to candidate central nervous system phenotypes. These include major heritable conditions, e.g., schizophrenia and other traits, e.g., behaviors that tend to increase and the motivation and ability to acquire and preserve social status of an individual. Our results showed that *ATRN* plays an important role in the testosterone level to affect the behavior. Schizophrenia patients with *ATRN* mutations have more proportion in abnormal testosterone level (Figures 5E,F), illustrating the relationship between *ATRN* and testosterone level. Finally, the impact of *Atrn* on maladaptive reward processing and other types of behavior sensitive to testosterone will need to be part of a future agenda.

## Data Availability Statement

The datasets presented in this study can be found in online repositories. The datasets Series record GSE178506 of transcriptome sequencing results for this study can be found in the GEO repository.

## Ethics Statement

The animal study was reviewed and approved by the IACUC of the Model Animal Research Center, Nanjing University. Written informed consent was obtained from the individual(s) for the publication of any potentially identifiable images or data included in this article. The studies involving human participants were reviewed and approved by the local ethics committee of the Affiliated Nanjing BrainHospital, Nanjing Medical University (2017-KY017). Written informed consent to participate in this study was provided by the legal guardian/next of kin of the participants.

## Author Contributions

XX, QZ, and NL contributed to the conception and design of the study. NL mainly performed the most experiments, organized the database, and wrote the first draft of the manuscript. SG collected the information and blood samples of patients. SW, SH, JW, LH, and DJ performed the statistical analysis and some experiments. YG, TC, and MK assisted in collecting samples. NL, YS, XX, and JZ participated in writing sections of the manuscript. All authors contributed to the manuscript revision and approved the submitted version.

## Conflict of Interest

The authors declare that the research was conducted in the absence of any commercial or financial relationships that could be construed as a potential conflict of interest.

## Publisher’s Note

All claims expressed in this article are solely those of the authors and do not necessarily represent those of their affiliated organizations, or those of the publisher, the editors and the reviewers. Any product that may be evaluated in this article, or claim that may be made by its manufacturer, is not guaranteed or endorsed by the publisher.

## Footnotes

1
http://crispr.mit.edu

## Abbreviations

MZ mut: maternal-zygote mutant
Dpf: days postfertilization
Mpf: months postfertilization
hATRN wt: human wild-type ATRN
HPG: hypothalamic–pituitary–gonad
LH: luteinizing hormone
PGC: primordial germ cells.
