# Supplementary material for: Attractin Participates in Schizophrenia by Affecting Testosterone Levels
Source: Front Cell Dev Biol. 2021 Nov 11;9:755165. doi: 10.3389/fcell.2021.755165 (PMC8636034; doi:10.3389/fcell.2021.755165)
Supplement: Supplementary file 1 [file Data_Sheet_1.pdf]

Supplementary Material

1 Supplementary Data

1.1 Supplementary Figures

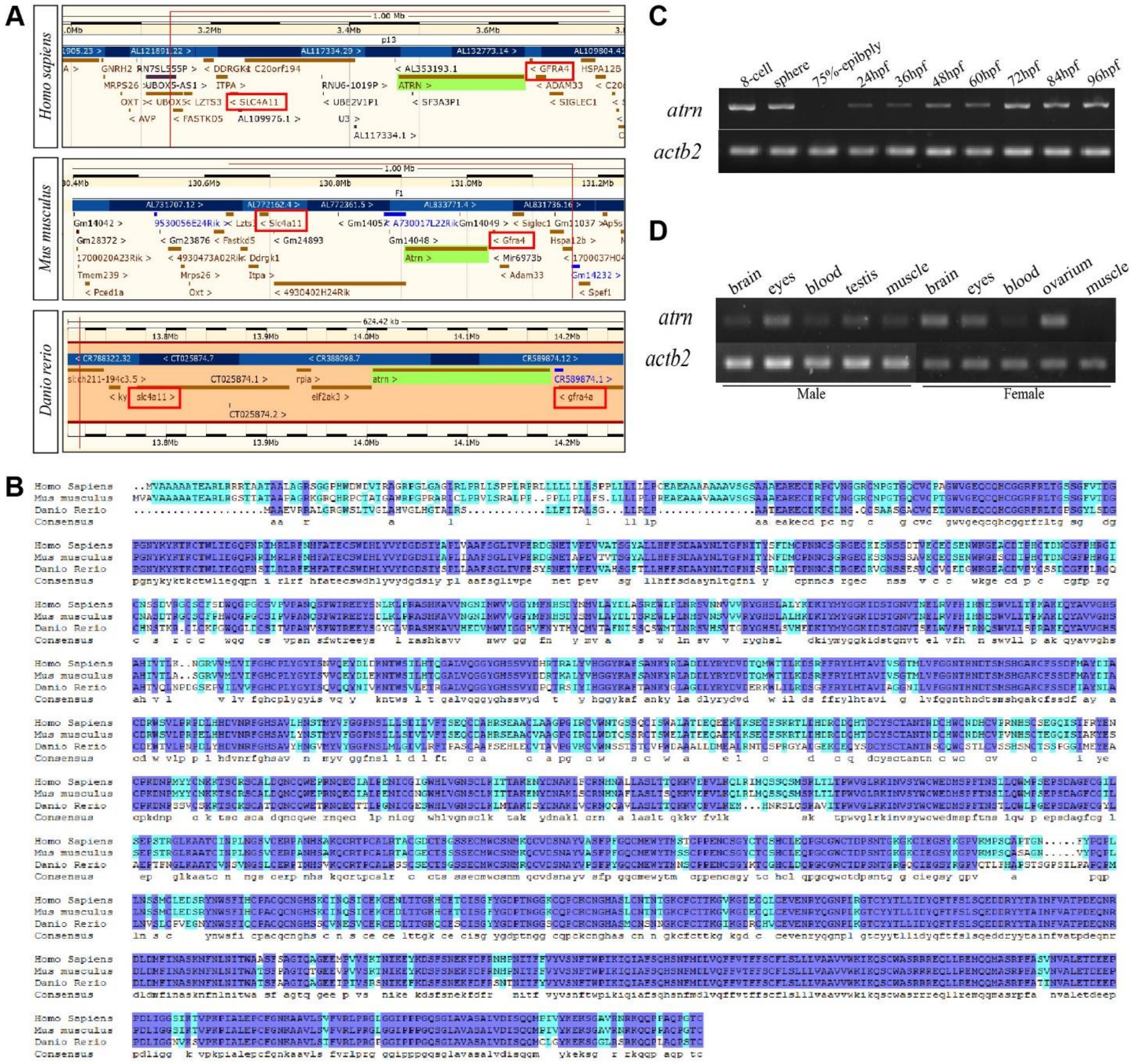

Supplementary Figure 1. Expression pattern of zebrafish ortholog gene atrn. (A) Syngeneic analysis of human, mice and zebrafish *ATRN* genes using the Ensembl genome browser (http://ensembl.org). In these three different species, there are two genes, *gfra4* and *slc4a11*, near the *ATRN* gene. (B) ATRN protein sequence alignment of human, mice and zebrafish attractin. (C) Semi-quantitative RT-

PCR detection of zebrafish *atrn* mRNA level at different development stages. (D) Semi-quantitative RT-PCR detection of zebrafish *atrn* mRNA level of adult zebrafish tissue.

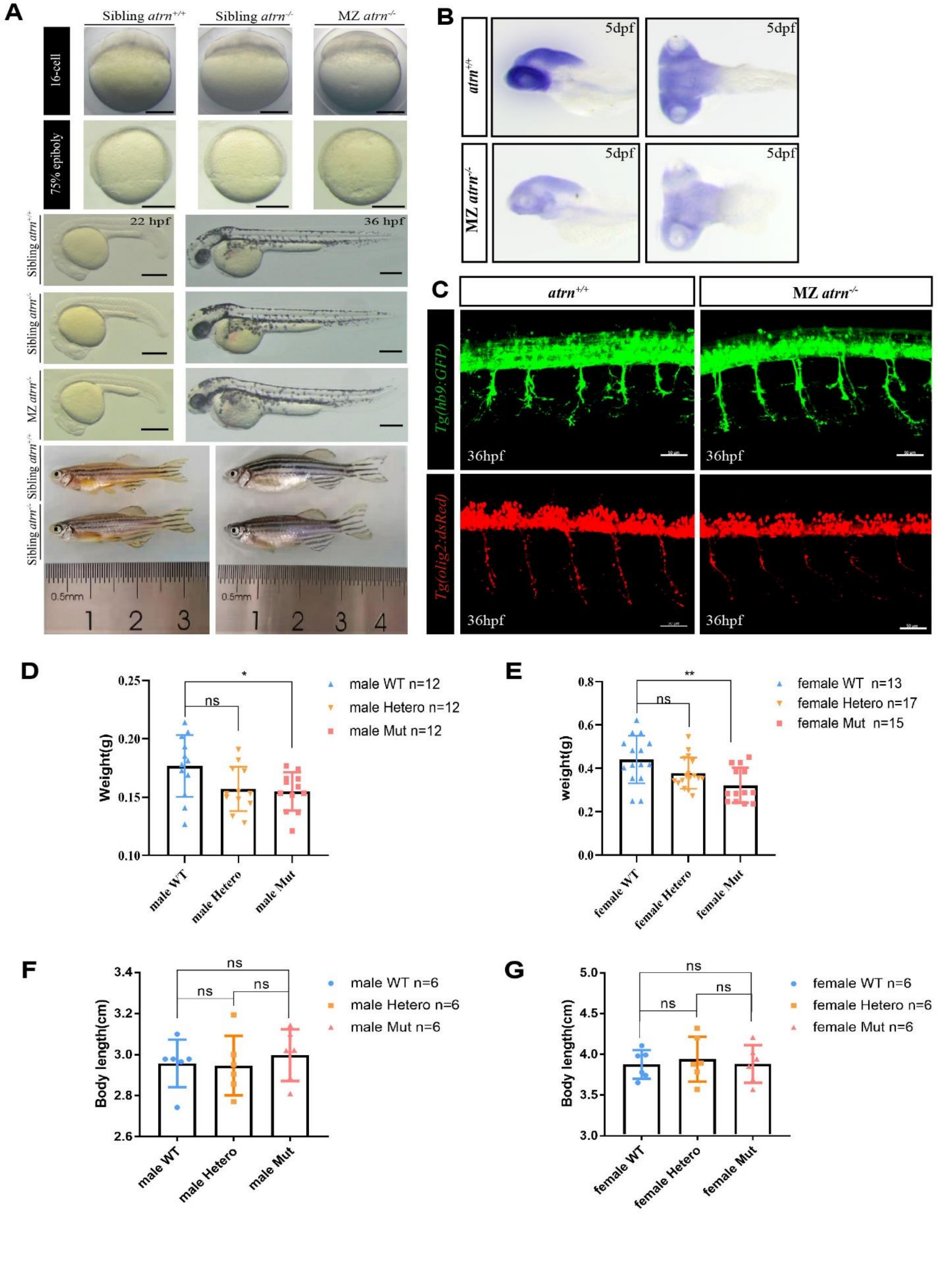
A
Sibling atrn+/+
Sibling atrn-/-
MZ atrn-/-
16-cell
75% epiboly
22 hpf
36 hpf
B
5dpf
atrn+/+
MZ atrn-/-
C
Tg(hb9:GFP)
Tg(olig2:dsRed)
36hpf
D
male WT n=12
male Hetero n=12
male Mut n=12
Weight(g)
E
female WT n=13
female Hetero n=17
female Mut n=15
weight(g)
F
male WT n=6
male Hetero n=6
male Mut n=6
Body length(cm)
G
female WT n=6
female Hetero n=6
female Mut n=6
Body length(cm)

Supplementary Figure 2. There are no obvious developmental defects in *atrn*$^{-/-}$. (A) The mutant can grow up normally with no obvious defects, and they can reproduce normally and lay eggs. (B) Reduced expression of *atrn* mRNA in *atrn*$^{+/+}$ and MZ *atrn*$^{-/-}$ larvae (5dpf) analyzed by WISH. (C) The development of motor neurons and oligodendrocytes was not affected in MZ *atrn*$^{-/-}$. (D) We measured the body weight of three genotype male adult zebrafishes, 12 in each group. The male mutant weigh much more than the wildtype and the heterozygous. (E) Identically, we measured 13 female *atrn*$^{+/+}$ zebrafishes, 17 female *atrn*$^{+/-}$ zebrafishes and 15 female *atrn*$^{-/-}$ zebrafishes, and the results as before in the male zebrafishes. (F-G) The length of different genotype adult zebrafish has no significantly difference. Data are shown as mean ± SD; **, $p<0.05$, **, $p<0.01$, ***, $p < 0.001$*.

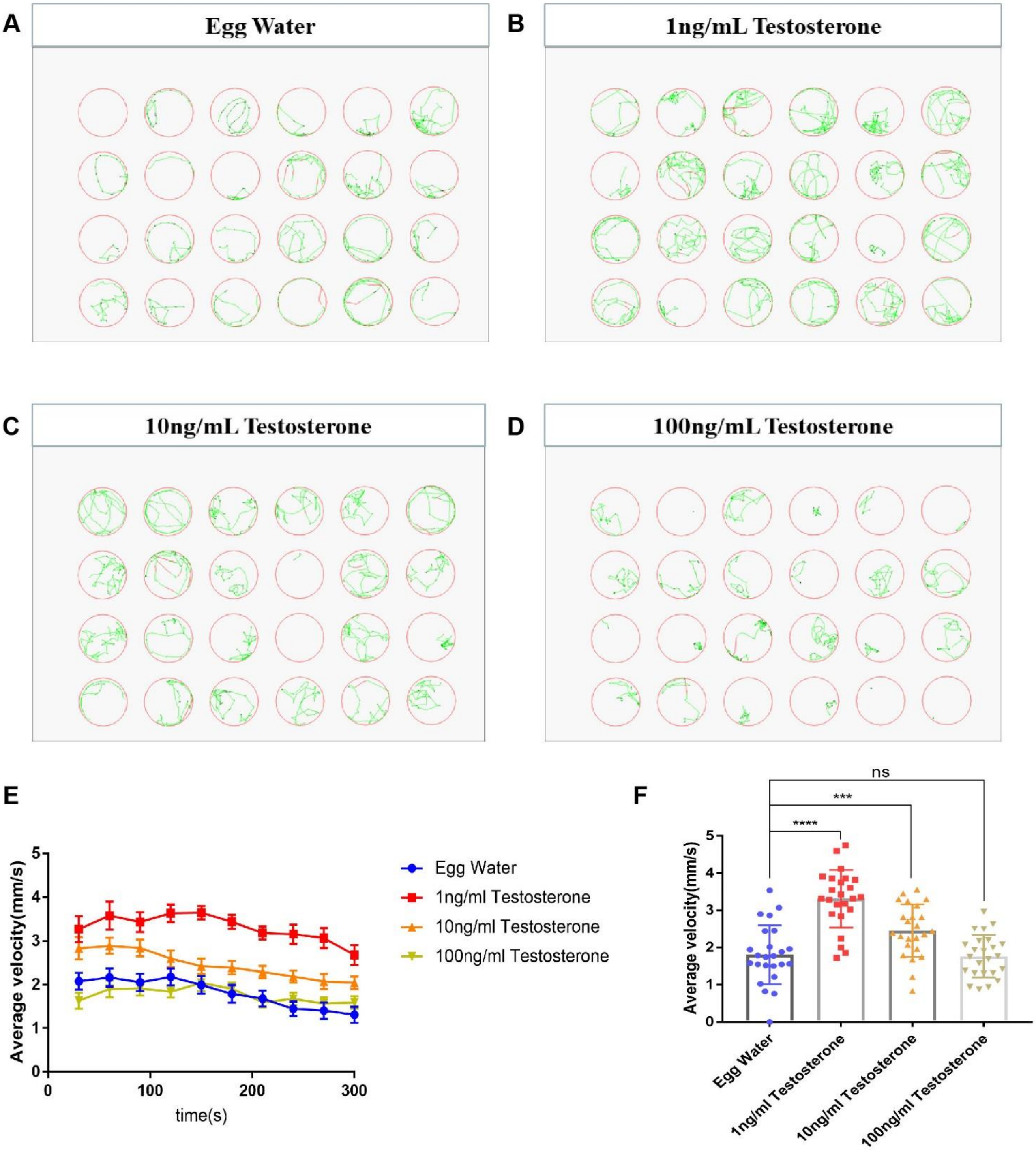

Supplementary Figure 3. The average velocity of external testosterone treated larvae is faster than the control. Locus diagram of the control group **(A)**, 1ng/mL testosterone treated group **(B)**, 10ng/mL testosterone treated group **(C)** and 100ng/mL testosterone treated group **(D)** at 5dpf. **(E)** The average velocity-time graph of testosterone treated larvae at 5dpf. (F) The average swimming speed of larvae during 5min period at 5dpf. Data are shown as mean ± SD; ****, $p<0.001$; ****, $p<0.0001$; ns, no significance ($p>0.05$).*

Table S1 Testosterone levels in men with mental disorders aged 20-49 years.

**Gender**	**Male (20-49)**					
	**Low T**	**Medium T**	**High T**	**Total**	**χ2, df**	***p value***
**Control**	5 (8.3%)	51 (85%)	4 (6.7%)	60		
**Epilepsy**	3 (14.2%)	17 (81%)	1 (4.8%)	21	0.6798, 2	0.7118
**Anxiety disorders**	9 (7.8%)	101 (87.8%)	5 (4.4%)	115	0.4612, 2	0.7941
**Schizophrenia**	215 (26.1%)	589 (71.4%)	21 (2.5%)	825	11.87, 2	0.0027**
**Obsessive-compulsive disorder**	2 (6.1%)	31 (93.9%)	0	33	2.539, 2	0.281
**Insomnia**	0	12 (100%)	0	12	2.057, 2	0.3575
**Bipolar disorder**	44 (22.2%)	141 (71.2%)	13 (6.6%)	198	5.854, 2	0.0536
**Depression disorder**	59 (11.4%)	442 (85.3%)	17 (3.3%)	518	2.15, 2	0.3413
**Undiagnosed mental disorder**	134 (19.5%)	516 (75.2%)	36 (5.3%)	686	4.616, 2	0.0994
**Mania**	14 (12.6%)	90 (81.1%)	7 (6.3%)	111	0.7223, 2	0.6969

*, $P < 0.05$; **, $P < 0.01$.

Table S2 Testosterone levels in women with mental disorders aged 20-49 years.

**Gender**	**Female (20-49)**					
	**Low T**	**Medium T**	**High T**	**Total**	**χ2, df**	***p value***

**Control**	8 (6.6%)	104 (86%)	9 (7.4%)	121		
**Epilepsy**	1 (2.9%)	33 (94.3%)	1 (2.9%)	35	1.767, 2	0.4134
**Anxiety disorders**	16 (8.1%)	159 (80.3%)	23 (11.6%)	198	1.813, 2	0.4039
**Schizophrenia**	42 (4.0%)	785 (75%)	219 (20.9%)	1046	13.51, 2	0.0012**
**Obsessive-compulsive disorder**	4 (19.0%)	17 (81%)	0	21	4.889, 2	0.0868
**Insomnia**	1 (4.0%)	24 (96%)	0	25	2.327, 2	0.3123
**Bipolar disorder**	16 (3.8%)	314 (74.4%)	92 (21.8%)	422	13.75, 2	0.001**
**Depression disorder**	96 (10.0%)	754 (78.4%)	112 (11.6%)	962	3.748, 2	0.1535
**Undiagnosed mental disorder**	56 (5.8%)	704 (73.1%)	203 (21.1%)	963	12.72, 2	0.0017**
**Mania**	5 (3.8%)	103 (77.4%)	25 (18.8%)	133	7.677, 2	0.0215*

*, $P < 0.05$; **, $P < 0.01$.

Table S3 Testosterone levels in men with mental disorders aged over 50 years.

**Gender**	**Male (>50)**					
	**Low T**	**Medium T**	**High T**	**Total**	***χ2, df***	***p value***
**Control**	7 (28.0%)	18 (72.0%)	0	25		
**Epilepsy**	1 (16.7%)	5 (83.3%)	0	6	0.3246, 1	0.5689

**Anxiety disorders**	2 (1.9%)	97 (90.7%)	8 (7.4%)	107	22.97, 2	<0.0001****
**Schizophrenia**	29 (12.6%)	193 (83.9%)	8 (3.5%)	230	5.04, 2	0.0805
**Obsessive-compulsive disorder**	0	1 (100%)	0	1	0.3832, 1	0.619
**Insomnia**	3 (9.1%)	26 (78.8%)	4 (12.1%)	33	6.067, 2	0.0482*
**Bipolar disorder**	11 (18.6%)	44 (74.6%)	4 (6.8%)	59	2.428, 2	0.297
**Depression disorder**	20 (5.7%)	303 (87.1%)	25 (7.2%)	348	18.37, 2	0.0001***
**Mental disorder**	23 (14.0%)	133 (81.1%)	8 (4.9%)	164	4.114, 2	0.1279
**Mania**	6 (16.7%)	26 (72.2%)	4 (11.1%)	36	3.667, 2	0.1598

*, $P < 0.05$; ***, $P < 0.001$; ****, $P < 0.0001$.

Table S4 Testosterone levels in men with mental disorders aged over 50 years.

**Gender**			**Female (>50)**			
	**Low T**	**Medium T**	**High T**	**Total**	**χ2, df**	***p value***
**Control**	6 (14.6%)	30 (73.2%)	5 (12.2%)	41		
**Epilepsy**	1 (25%)	3 (75%)	0	4	0.7412, 2	0.6903
**Anxiety disorders**	21 (9.6%)	190 (87.2%)	7 (3.2%)	218	7.634, 2	0.022*
**Schizophrenia**	33 (8.9%)	305 (82.4%)	32 (8.6%)	370	2.175, 2	0.3371

**Obsessive-compulsive disorder**	0	7 (87.5%)	1 (12.5%)	8	1.353, 2	0.5083
**Insomnia**	6 (12.5%)	42 (87.5%)	0	48	6.49, 2	0.039*
**Bipolar disorder**	11 (10.7%)	89 (86.4%)	3 (2.9%)	103	5.559, 2	0.0621
**Depression disorder**	69 (9.8%)	596 (84.7%)	39 (5.5%)	704	4.413, 2	0.1101
**Mental disorder**	28 (12.2%)	186 (81.2%)	15 (6.6%)	229	1.938, 2	0.3795
**Mania**	2 (12.5%)	11 (68.8%)	3 (18.8%)	16	0.4209, 2	0.8102

*$P < 0.05$.

Table S5 Testosterone levels of 14 ATRN-mutated schizophrenia patients were tested at different time points.

Patients	Gender	Age	Test Date	Testosterone (ng/ml)	Testosterone (nmol/L)	Change
1	Female	49	2010/8/11	0.1600	0.5024	High
2	Male	40	2011/5/27	0.42	1.3188	Low
3	Female	43	2011/10/8	0.28	0.8792	High
4	Female	36	2013/10/22	0.162	0.50868	High
5	Female	57	2019/5/16	0.216	0.67824	High
6	Female	32	2013/10/23	0.389	1.22146	High
7	Female	34	2018/5/15	0.402	1.26228	High
8	Male	20	2013/3/6	3.68	11.5552	--
9	Female	36	2014/2/24	0.362	1.13668	--

10	Female	48	2016/10/21	0.239	0.75046	--
11	Male	25	2019/1/24	4.4236	13.890104	--
12	Male	17	2018/11/6	7.0288	22.070432	--
13	Female	42	2011/10/13	0.89	2.7946	High
14	Male	28	2018/6/20	0.257	0.80698	Low

High: Testosterone level is higher than the normal range; Low: Testosterone level is lower than the normal range; --: Testosterone level is within the normal range.
